# Evaluating The Effect of Melatonin on *HAS2* , and *PGR* Expression, as Well as Cumulus Expansion, and Fertility Potential in Mice

**DOI:** 10.22074/cellj.2018.4894

**Published:** 2018-01-01

**Authors:** Maryam Ezzati, Leila Roshangar, Jafar Soleimani Rad, Nahid Karimian

**Affiliations:** 1 Stem Cell Research Center, Tabriz University of Medical Sciences, Tabriz, Iran; 2 Department of Anatomical Sciences, Faculty of Medicine, Tabriz University of Medical Sciences, Tabriz, Iran; 3 Department of Tissue Engineering, Faculty of Medicine, Tabriz University of Medical Sciences, Tabriz, Iran; 4 Department of Advanced Sciences, Faculty of Medicine, Tabriz University of Medical Sciences, Tabriz, Iran

**Keywords:** Hyaluronan Synthase-2, Melatonin, Mouse Oocyte, Progesterone Receptor

## Abstract

**Objective:**

Infertility is a worldwide health problem which affects approximately 15% of sexually active couples. One of the factors influencing the fertility is melatonin. Also, protection of oocytes and embryos from oxidative stress inducing chemicals in the culture medium is important. The aim of the present study was to investigate if melatonin could regulate hyaluronan synthase-2 (*HAS2* ) and Progesterone receptor (*PGR* ) expressions in the cumulus cells of mice oocytes and provide an *in vitro* fertilization (IVF) approach.

**Materials and Methods:**

In this experimental study, for this purpose, 30 adult female mice and 15 adult male mice were used. The female mice were superovulated using 10 U of pregnant mare serum gonadotropin (PMSG) and 24 hours later, 10 U of human chorionic gonadotropin (hCG) were injected. Next, cumulus oocyte complexes (COCs) were collected from the oviducts of the female mice by using a matrix-flushing method. The cumulus cells were cultured with melatonin 10 µM for 6 hours and for real-time reverse transcription-polymerase chain reaction (RT-PCR) was used for evaluation of *HAS2* and *PGR* expression levels. The fertilization rate was evaluated through IVF. All the data were analyzed using a t test.

**Results:**

The results of this study showed that *HAS2* and *PGR* expressions in the cumulus cells of the mice receiving melatonin increased in comparison to the control groups. Also, IVF results revealed an enhancement in fertilization rate in the experimental groups compared to the control groups.

**Conclusion:**

To improve the oocyte quality and provide new approaches for infertility treatment, administration of melatonin as an antioxidant, showed promising results. Thus, it is concluded that fertility outcomes can be improved by melatonin it enhances *PGR* .

## Introduction

As defined by world health organization (WHO) infertility is as the inability of a couple of child-bearing age to conceive over 12 months of regular unprotected sexual intercourse and is considered as a public health problem (1). About 10-15% of young couples have been reported to suffer from this conditions. Of these, 40-55, 20-30, and 15-17% are due to female factors, male factors and unexplained conditions, respectively (2, 3). 

For 10% of couples trying to conceive underlying causeof the infertility are not easily identifiable, though all theirrelevant tests are normal. In this case, *in vitro* fertilization (IVF) may be recommended since it has shown to bethe most effective treatment for unexplained infertility(4). Conditions such as exposure to light, elevated oxygen concentrations, and unusual concentrations of metabolites can cause an oxidative stress in oocytes and embryos during *in vitro* culture. Reactive oxygen species (ROS) can damage cell membranes and DNA and cause apoptosis (5). Therefore, protecting oocytes and embryos from oxidative stress in the culture medium is of crucial importance. In this regard, the usage of antioxidant compounds may result in more favorable results. 

Oxidative stress can be reduced and culture conditions can be promoted an antioxidant or a radical scavenger is added to in the *in vitro* culture medium. As the main hormone secreted by pineal gland in the human brain, melatonin (N-acetyl-5methoxytryptamine) is an endogenous compound which was discovered about 50 years ago. Through its stimulatory actions on the antioxidant system, it is regarded as a direct free-radical scavenger and an indirect antioxidant (6-8). 

Several studies have demonstrated that melatonin is a powerful direct free-radical scavenger. In contrast to a majority of other known radical scavengers, melatonin is a universal multifunctional antioxidant. Melatonin has been reported to act as a hydrophilic and hydrophobic antioxidant since it is both lipid and water soluble (9). Also, the presence of melatonin receptors in the ovary at multiple sites, indicates that melatonin may influence the reproductive system (10). Melatonin concentrations in ovarian follicles increase with follicular growth (11). Furthermore, melatonin treatmentenhances the hCG-stimulated progesterone secretion (12). Earlier studies indicated that progesterone, a steroidhormone is a key player in ovulation. The biological effectsof progesterone are mediated via *PGR* , a ligand-activated transcription factor. Ovarian *PGR* expression is undetectableduring follicular development (13). It is likely that some *PGR* - regulated factors are secreted by granulosa cells and deliveredto other cell types, such as cumulus cells within the ovary. Furthermore, besides playing a critical role in the ovary, progesterone is known to be essential for the maintenance of pregnancy. *PGR* s as members of the nuclear receptorsuperfamily of transcription factors (NR3C3) mediate progesterone effects (14).

*PGR* has been identified as an important regulator of genetranscription during the peri-ovulatory period, especially*PGR* regulates the transcription of the genes required for asuccessful oocyte release from the preovulatory follicle. In response to luteinizing hormone (LH), granulosa cellsrelease epidermal growth factor (EGF)-like ligands, whichin turn, cause cumulus cells to undergo an expansion (15). The cumulus oocyte complexes (COCs) formed due to thepresence of a unique extracellular matrix plays an importantrole in successful oocyte maturation and ovulation (16). Atthe same time, the oocyte resumes meiosis and undergoesa maturation process to become competent for ovulation, fertilization, and expansion of COCs. During the expansionof cumulus cells, *HAS2* is the most important gene involved in the production of hyaluronic acid (HA) matrix (17). 

It is generally accepted that *HAS2* mRNA is a key elementrequired for the cumulus expansion process, which is necessaryfor oocyte maturation and ovulation (17-21). Increasing cumulus cell expansion can improve fertility rates because *HAS2* and *PGR* expression rates are enhanced during cumulus expansion. Melatonin may influence the cumulus expansionby augmenting *HAS2* and *PGR* expressions. Therefore, the effects of melatonin on *HAS2* and *PGR* expression and cumulus expansion were evaluated in this study.

## Materials and Methods

This experimental study was approved by the Research Committee of Tabriz Medical University according to the rules of the Ethics Committee. Forty five adult mice (15 male and 30 females, 30-35 g and 6-8 weeks old) were obtained from the animal house of Tabriz University of Medical Sciences (TUMS). Animals were kept in a 12-hour light/12hour dark cycle with an unrestricted access to food and water at room temperature for 2 weeks.

Preparation of melatonin stock solution was done using an ethanol/TCM199 system. For this purpose 23.23 mg of melatonin (Sigma, United states) was dissolved in 1 ml of 0.1% absolute ethanol and diluted with TCM199 and serial dilutions were prepared. Using this method, a stock solution of melatonin 10 µM was prepared (22) and stored in a refrigerator at 4°C at most for 2 weeks. In our experiment, ethanol amount was 0.1% in the maturation medium.

All the animals were treated in accordance with the guidelines of University Ethics Committee for care and use of laboratory animals. The female mice were superovulated using an intraperitoneal injection of 10 IU human menopausal gonadotropin (HMG) (NV Organon, Oss, The Netherlands) and after 24 hours, another intraperitoneal injection of 10 IU of human chorionic gonadotropin (hCG) (NV Organon, Oss, Holland) was done.

The female mice were sacrificed within 48 hours of hCG injection, and the ovaries and oviducts were removed after. Next, the mice ovaries (n=60) and oviducts were placed in sterile phosphate-buffered saline (PBS, Sigma, USA). Then, samples were transported to the Tissue Engineering Laboratory. Upon removing the stromal tissues surrounding the oviducts, the oocytes were collected from the uterine tube using the flushing method (universal medium of Azar Panam). By using a head sampler, the oocytes were drawn and poured into a dish. Finally liquid oil was added to prevent the culture medium evaporation.

The cumulus cells surrounding the oocytes, (i.e., cumulus oocyte complexes (COCs) were transferred to another dish containing the medium and then PBS was added for washing. In the experimental groups, the cumulus cells were cultured in the medium supplement with 10 µM melatonin for 6 hours. For evaluation of cumulus expansion, COCs were morphologically classified at recovery as having a compact or expanded investment. COCs were cultured in 200 mµ of a universal IVF medium and then incubated with sterile mineral oil at 37°C with 5% CO_2_ for 6 hours. After this incubation period, the medium was centrifuged twice at 3000 rpm for 5 minutes. Afterwards, the pellet of the cumulus cells was transferred to -20°C for 1 hour and then stored at -80°C until RNA isolation.

### *In vitro* fertilization 

The animals selected for IVF were divided into control and experimental groups. After superovulation, the femalemice were killed by cervical dislocation, their oocytes were collected by uterine tube flushing. Next, sperms were collectedfrom the caudal epididymis of male mice by incubating the pieces of epididymis with Ham’s F-10 medium cultureusing a CO_2_ incubator at 37°C for 20 minutes. The spermsamples were added to the collected oocytes of the controland experimental groups. The rate of fertility success was evaluated based on embryo formation associated with more cleavages and morula.

### Real time reverse transcription-polymerase chain reaction 

To measure *HAS2* and *PGR* mRNA in the control and experimental groups, RNA expression was determined by using real time reverse transcription-polymerase chainreaction (RT-PCR) assay. The primers used in the PCR are presented in Table 1. Glyceraldehyde-3-phosphatedehydrogenase (GAPDH) was used as the internal controlgene to normalize the results. By using RNeasy Micro kit(CinnaGen, Iran), the total RNAs of *HAS2* and PRs were extracted. Primers were designed and real-time RT-PCR wasconducted to analyze the gene expressions using SYBR green technology.

**Table 1:** Primer process for real-time reverse transcription-polymerase chain reaction


Gene	Sequence (5ˊ-3ˊ)	Start	Stop	Product length

*HAS2*	F: CCTCACTGCGCAGACTACCA	3876	3895	131
	R: CCATACGGCGAGAGTCGGAG	4006	3987	
*PGR*	F: TGTTGTCAGGCTGGCATGGT	2493	2512	182
	R: AGTGGCGGGACCAGTTGAAT	2674	2655	
*GAPDH*	F: CGGGGTCCCAGCTTAGGTTC	30	49	103
	R: GCCCAATACGGCCAAATCCG	132	113	


### Statistical analysis 

Using SPSS software, version 22 and the sample t-test, all the statistical analyses were performed. P<0.05 were considered statistically significant. 

## Results

### Effect of melatonin on *HAS2* and *PGR* expression in cumulus cells

**Fig.1:**
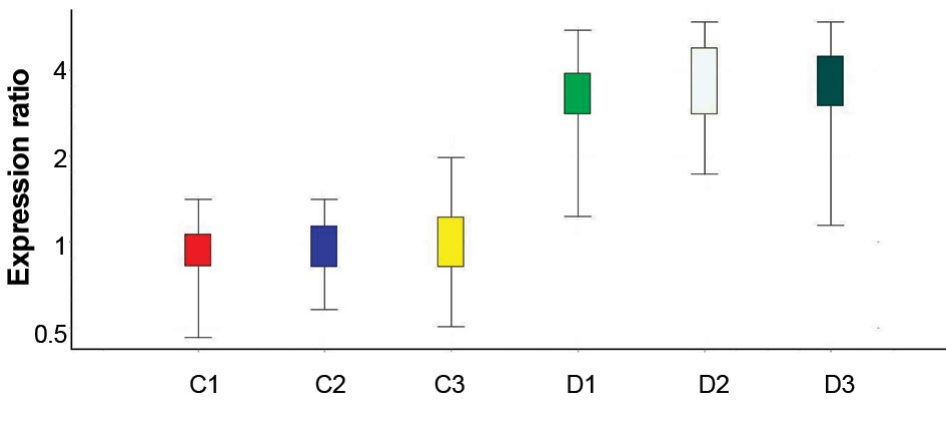
*PGR* gene expression in isolated cumulus cell in control groups (C) and in groups received melatonin (D).

**Fig.2:**
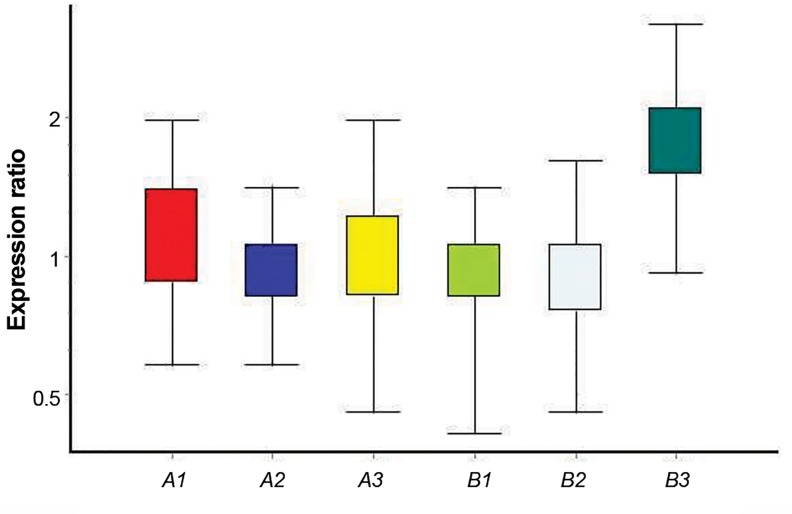
*HAS2* gene expression in isolated cumulus cell in control groups (A) and in groups received melatonin (B).

*HAS2* and *PGR* mRNA expressions in cumulus cells isolated from mice oocytes were evaluated using quantitative real-time RT-PCR. A significant increase was observed in *PGR* mRNA in the experimental compared to the control group (Fig .1). However, no significant differences in *HAS2* gene expression were observed between the experimental and control groups (Fig .2). At this stage, each of the control and experimental groups were processed for real-time RTPCR assay for 3 times. Hence, 3 experimental and 3 control groups are shown for each gene in the diagram (Figes.1, 2).

### Melatonin effect on fertilization rate and cumulus expansion

**Fig.3:**
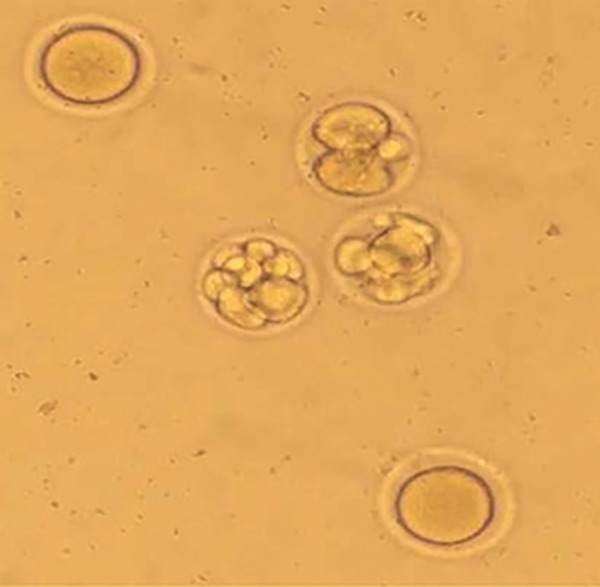
A photomicrograph of inseminated oocytes in melatonin-treated mice.

**Fig.4:**
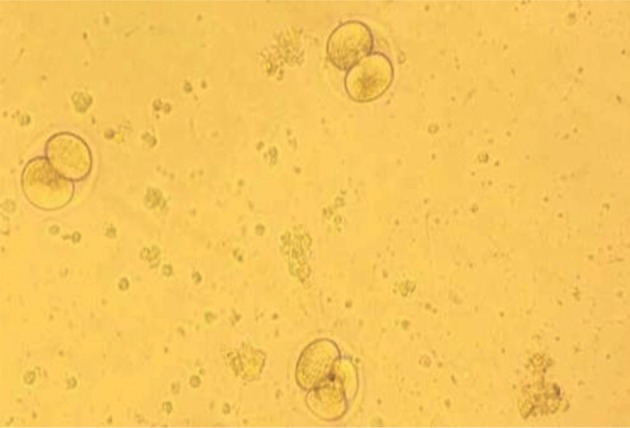
A photomicrograph of inseminated oocytes in control group.

IVF was carried out in both control and experimental groups and 100 oocytes were evaluated from each group. By counting the number of embryos, it was revealed that more oocytes were developed into the embryos in the experimental group receiving melatonin for 6 hours as compared to the control group which did not receive melatonin (P<0.05). The embryos developed in the experimental and control groups are shown in Figures 3 and 4, respectively. Moreover, compact cumulus was seen to be tightly attached to the cells surrounding the smooth-surfaced oocytes over the cumulus hillock as shown by evaluating of the uniformity of ooplasm. On the other hand, expanded cumulus cells were detached from the oocytes with a matrix visible between the cumulus cells. The numbers of the expanded and compact COCs are demonstrated in Table 2. The statistical analysis showed that embryo formation in the experimental group was significantly increased (P<0.05, Table 2). 

**Table 2:** Embryo formation percentage in the control and experimental groups


Oocyte	Experimental Group (%)	Control Group (%)	P value

Fertilization	80	77	<0.05
Oocyte expansion rate	64	36	<0.05


## Discussion

It is generally accepted that melatonin and the presence of its receptors in granulosa cells can potentially exert beneficial effects on the ovarian function (22). As melatonin is a powerful free-radicals scavenger which is even more potent than vitamin E (23), it might be able to protect granulosa cells from the cytotoxicity of free- radicals that could be produced following long-term in vitro culture (24, 25). 

The results of the present research are in agreement with previous histological and immunohistochemical studies, which showed that free-radicals scavenger could promote the qualities of the cumulus-oocyte complexes through inducing a uniform distribution of follicle cells covering the oocytes in the ovaries (26). These findings are in line with those reported the study of Bahadori et al. (9), describing the detrimental effect of oxidative stress on oocyte microenvironment and subsequently on implantation, follicular development, ovulation, oocyte quality, and early embryonic development. Also, the results of the study conducted by Ishizuka et al. (27) is consistent with those of the current research indicating that melatonin supports both mice fertilization and early development of embryonic tissue in the culture medium. 

However, no relationships between oxidative stress markers and pregnancy rate were reported by Jozwick et al. (28) *HAS2* gene expression in cumulus cells is critical for the formation of hyaluronan, the predominant matrix component surrounding the expanded cumulus cells. Hyaluronan synthesis, which appears to occur at the transcription level of *HAS2* , is strictly regulated in cumulus cells. The present study confirmed the role of melatonin in promoting cumulus cell expression of *HAS2* though the influence was not significant (29-31). The results of another study showed that oocyte quality is ameliorated by antioxidants, while it directly increases the degrees of cumulus cell expansion and *HAS2* expression (32). Moreover, observed in our study, it was previously shown that in that melatonin increases the number of *PGR* s (33). The present study revealed the effects of administration of melatonin on IVF, cumulus cell expansion, maturation, and *HAS2* and *PGR* expressions.

## Conclusion

It is concluded that melatonin can improve IVF outcome by increasing cumulus cell expansion and *PGR* expression, while it had no influence on *HAS2* expression.

## References

[1] Araoye MO (2003). Epidemiology of infertility: social problems of the infertile couples. West Afr J Med.

[2] Bower C, Hansen M (2005). Assisted reproductive technologies and birth outcomes: overview of recent systematic reviews. Reprod Fertil Dev.

[3] Talaulikar VS, Arulkumaran S (2013). Maternal, perinatal and long-term outcomes after assisted reproductive techniques (ART): implications for clinical practice. Eur J Obstet Gynecol, Reprod Biol.

[4] Society for Assisted Reproductive Technology; American Society for Reproductive Medicine (2004). Assisted reproductive technology in the United States: 2000 results generated from the American Society for Reproductive Medicine/Society for Assisted Reproductive Technology Registry. Fertil Steril.

[5] Agarwal A, Said TM, Bedaiwy MA, Banerjee J, Alvarez JG (2006). Oxidative stress in an assisted reproductive techniques setting. Fertil Steril.

[6] Hardeland R (2005). Antioxidative protection by melatonin: multiplicity of mechanisms from radical detoxification to radical avoidanc. Endocrine.

[7] Polat MF, Taysi S, Gul M, Cikman O, Yilmaz I, Bakan E (2002). Oxidant/antioxidant status in blood of patients with malignant breast tumour and benign breast disease. Cell Biochem Funct.

[8] Sharma S, Haldar C, Chaube SK (2008). Effect of exogenous melatonin on X-ray induced cellular toxicity in lymphatic tissue of Indian tropical male squirrel, Funambulus pennanti. Int J Radiat Biol.

[9] Bahadori MH, Ghasemian F, Ramezani M, Asgari Z (2013). Melatonin effect during different maturation stages of oocyte and subsequent embryo development in mice. Iran J Reprod Med.

[10] Woo MM, Tai CJ, Kang SK, Nathwani PS, Pang SF, Leung PC (2001). Direct action of melatonin in human granulosa-luteal cells. J Clin Endocrinol Metab.

[11] Tamura H, Takasaki A, Taketani T, Tanabe M, Kizuka F, Lee L (2012). The role of melatonin as an antioxidant in the follicle. J Ovarian Res.

[12] Ekmekcioglu C (2006). Melatonin receptors in humans: biological role and clinical relevance. Biomed Pharmacother.

[13] Park OK, Mayo KE (1991). Transient expression of progesterone receptor messenger RNA in ovarian granuiosa cells after the preovulatory luteinizing hormone surge. Mol Endocrinol.

[14] Tsai MJ, O’Malley BW (1994). Molecular mechanisms of action of steroid/ thyroid receptor superfamily members. Annu Rev Biochem.

[15] Park JY, Su YQ, Ariga M, Law E, Jin SL, Conti M (2004). EGF-like growth factors as mediators of LH action in the ovulatory follicle. Science.

[16] Russell DL, Robker RL (2007). Molecular mechanisms of ovulation: coordination through the cumulus complex. Hum Reprod Update.

[17] Sugiura K, Su YQ, Eppig JJ (2009). Targeted suppression of Has2 mRNA in mouse cumulus cell-oocyte complexes by adenovirus-mediated short-hairpin RNA expression. Mol Reprod Dev.

[18] Adriaenssens T, Segers I, Wathlet S, Smitz J (2011). The cumulus cell gene expression profile of oocytes with different nuclear maturity and potential for blastocyst formation. J Assist Reprod Genet.

[19] Curry TE Jr (2010). ADAMTS1 and versican: partners in ovulation and fertilization. Biol Reprod.

[20] Gebhardt KM, Feil DK, Dunning KR, Lane M, Russell DL (2011). Human cumulus cell gene expression as a biomarker of pregnancy outcome after single embryo transfer. Fertil Steril.

[21] Hong SJ, Chiu PC, Lee KF, Tse JY, Ho PC, Yeung WS (2009). Cumulus cells and their extracellular matrix affect the quality of the spermatozoa penetrating the cumulus mass. Fertil Steril.

[22] Adriaens I, Jacquet P, Cortvrindt R, Janssen K, Smitz J (2006). Melatonin has dose-dependent effects on folliculogenesis, oocyte maturation capacity and steroidogenesis. Toxicology.

[23] Tan DX, Chen LD, Poeggeler B, Manchester LC, Reiter RJ (1993). Melatonin: a potent, endogenous hydroxyl radical scavenger. Endocr J.

[24] Rodriguez C, Mayo JC, Sainz RM, Antolin I, Herrera F, Martin V (2004). Regulation of antioxidant enzymes: a significant role for melatonin. J Pineal Res.

[25] Reiter RJ, Acuña-Castroviejo D, Tan DX, Burkhardt S (2001). Free radicalmediated molecular damage.Mechanisms for the protective actions of melatonin in the central nervous system. Ann N Y Acad Sci.

[26] Roushangar L, Rad JS (2007). Ultrastructural alterations and occurrence of apoptosis in developing follicles exposed to low frequency electromagnetic field in rat ovary. Pak J Biol Sci.

[27] Ishizuka B, Kuribayashi Y, Murai K, Amemiya A, Itoh MT (2000). The effect of melatonin on in vitro fertilization and embryo development in mice. J Pineal Res.

[28] Jozwik M, Wolczynski S, Jozwik M, Szamatowicz M (1999). Oxidative stress markers in preovulatory follicular fluid in humans. Mol Hum Reprod.

[29] Fülöp C, Salustri A, Hascall VC (1997). Coding sequence of a hyaluronan synthase homologue expressed during expansion of the mouse cumulus-oocyte complex. Arch Biochem Biophy.

[30] Elvin JA, Clark AT, Wang P, Wolfman NM, Matzuk MM (1999). Paracrine actions of growth differentiation factor-9 in the mammalian ovary. Mol Endocrinol.

[31] Dragovic RA, Ritter LJ, Schulz SJ, Amato F, Armstrong DT, Gilchrist RB (2005). Role of oocyte-secreted growth differentiation factor 9 in the regulation of mouse cumulus expansion. Endocrinology.

[32] Namvar Vansofla F, Roshangar L, Montaseri A, Soleimani Rad J (2016). Impact of prunus cerasus on PGR and HAS2 in cumulus cells and fertility outcome. Adv Pharm Bull.

[33] Abd-Allah AR, El-Sayed el SM, Abdel-Wahab MH, Hamada FM (2003). Effect of melatonin on estrogen and progesterone receptors in relation to uterine contraction in rats. Pharmacol Res.

